# Visual Loss Following Indirect Trauma: A Diagnostic Dilemma

**DOI:** 10.7759/cureus.104274

**Published:** 2026-02-26

**Authors:** Aini Zahidah Ismail, Hun Heng Tan, Evelyn Li Min Tai, Abirami Shavani Sanmugam, Rohana Taharin

**Affiliations:** 1 Ophthalmology and Visual Sciences, School of Medical Sciences, Universiti Sains Malaysia, Kubang Kerian, MYS; 2 Ophthalmology, Hospital Bukit Mertajam, Bukit Mertajam, MYS

**Keywords:** indirect trauma, optical coherent tomography, paracentral acute middle maculopathy, purtscher’s retinopathy, traumatic optic neuropathy

## Abstract

Monocular vision loss from indirect trauma can result in damage to the optic nerve, retina, or surrounding structures, often occurring without direct injury to the eye. In some cases, overlapping conditions can complicate the diagnosis. We present a case of monocular vision loss following indirect trauma, caused by traumatic optic neuropathy (TON) and Purtscher's retinopathy, emphasizing the diagnostic challenges encountered. A 28-year-old gentleman with no underlying medical conditions, who was involved in a motor vehicle accident and developed a sudden onset of central scotoma in his left eye three days later. His left visual acuity was hand movement, and 6/6 in the right eye. Left eye presented a positive relative afferent pupillary defect (RAPD), with reduced red saturation and light brightness. Anterior segment and intraocular pressure were unremarkable. Dilated fundus examination showed disc hemorrhage, peripapillary cotton wool spots (CWS), and swollen retinal nerve fibre layers surrounding the disc. Minimal dot-blot hemorrhage seen at the macula. Amsler’s grid and Bjerrum visual field test showed central scotoma. Optical coherence tomography (OCT) showed retinal thickening and oedema, with a hyperreflective band at the inner nuclear layer and at the perifoveal region, like paracentral acute middle maculopathy (PAMM). The right eye was unremarkable. Contrast computed tomography (CT) of the brain and orbit ruled out intracranial bleeding, cerebral oedema, and skull fracture. The patient was initially treated as left TON, as no other associated injuries with potential embolic etiologies, such as long bone fractures or chest compressions, suggested Purtscher’s retinopathy. He was treated with a three-day course of megadose intravenous methylprednisolone. On day 2 of treatment, left eye visual acuity improved to 6/18. We then re-evaluated OCT and noticed PAMM, which is suggestive of Purtscher’s retinopathy. Subsequently, his left visual acuity regained 6/6. Peripapillary CWS and hemorrhages diminished, with improvement seen on OCT and Bjerrum. This case highlights the clinical presentation of indirect TON combined with Purtscher’s retinopathy. Multimodal imaging, such as OCT, provides more information, which is helpful in diagnosing Purtscher’s retinopathy. Early recognition and management are crucial in optimizing visual outcomes and addressing the complex nature of such traumatic injuries.

## Introduction

Purtscher’s retinopathy refers to a chorioretinopathy associated with indirect trauma to the eye, characterized by funduscopic appearance with cotton wool spots (CWS), retinal hemorrhages, optic disc edema, and Purtscher flecken (areas of inner retinal whitening), associated with reduced visual acuity [1]. It is also known as traumatic retinal angiopathy or lymphorrhagia retinae or retinal teletraumatism, which is an occlusive microvasculopathy [2].

Traumatic optic neuropathy (TON) is a type of optic nerve injury that occurs secondary to trauma and has been etiologically associated with acute axonal loss with severe vision loss [3]. Indirect TON refers to a variation of TON that is caused by forces transmitted at a distance from the optic nerve after blunt force trauma to the head [3], and it is thought to be the result of a shock that has been transmitted from an orbital impact to the intracanalicular portion of the optic nerve. However, structural damage may first be detected in the macula and peripapillary area [4].

We present a case of Purtscher’s retinopathy with TON in a patient who experienced indirect trauma. This patient had delayed unilateral poor vision, which started three days post-mild head injury. Left relative afferent pupillary defect (RAPD) was positive, which raised suspicion of indirect TON, as his CT brain and orbit were normal. However, his fundus and optical coherence tomography (OCT) findings were consistent with Purtscher’s retinopathy features. His visual recovery was good after megadose steroid treatment. OCT on subsequent follow-ups showed improvement in retinal thickness. We conclude that this patient may have Purtscher’s retinopathy in the setting of TON.

This case was presented earlier as an e-poster (PO-113) during the 38th Asia-Pacific Academy of Ophthalmology (APAO) Congress in conjunction with the 13th Malaysian Society of Ophthalmology (MSO) Annual Scientific Meeting 2023 in Kuala Lumpur [5].

## Case presentation

A 28-year-old gentleman with no comorbidities presented with a complaint of a sudden onset of central scotoma over the left eye, which he experienced only three days after he was involved in a motor vehicle accident. He was a motorbike rider who had hit the back of a car and sustained a small laceration wound over his forehead. He had no loss of consciousness, retrograde amnesia, severe head trauma, or any bone fractures post injury.

**Figure 1:**
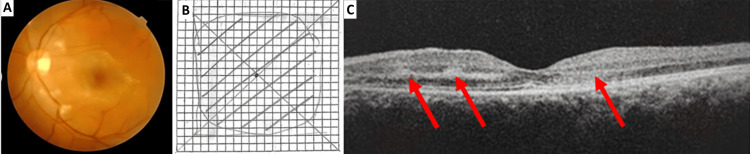
Left eye, three days post-trauma at presentation. (A) Fundus photograph. (B) Amsler grid test. (C) Optical coherence tomography (OCT) of the macula (red arrows).

His visual acuity was hand movement in the left eye and 6/6 in the right eye. He had a left RAPD, with reduced red saturation and light brightness. Dilated fundus examination of the left eye showed disc hemorrhages, peripapillary CWS, and retinal nerve fibre layer swelling, as well as dot-blot hemorrhages involving the macula (Figure 1A). The right eye fundus was normal. The anterior segment and intraocular pressures were unremarkable bilaterally. Amsler grid test confirmed a large central scotoma over the left eye (Figure 1B). OCT of the left eye macula revealed retinal thickening and oedema, with a hyperreflective band at the inner nuclear layer and the perifoveal region, suggestive of PAMM (red arrows) (Figure 1C). Contrast computed tomography (CT) of the brain and orbit was negative for fractures, intracranial bleeding, or cerebral oedema.

**Figure 2:**
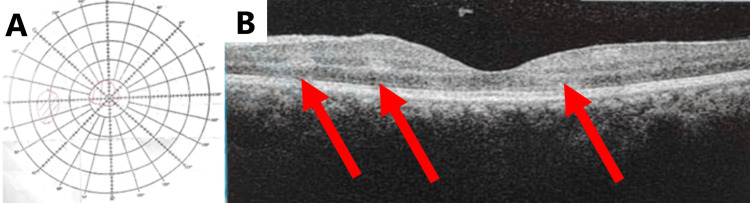
Left eye, day 2 of treatment. (A) Bjerrum visual field test. (B) Optical coherence tomography (OCT) of the macula (red arrows).

The patient was treated with a three-day course of megadose intravenous methylprednisolone 1 g OD. On day 2 of treatment, the left eye visual acuity improved to 6/18 and returned to 6/6 by day 3 of treatment. Oral prednisolone 60 mg OD was continued to complete 14 days of total steroid treatment. Bjerrum visual field test showed a reduction of central scotoma (Figure 2A), and OCT macula revealed slight improvement of retinal thickening and paracentral acute middle maculopathy (PAMM) (red arrows) (Figure 2B).

**Figure 3:**
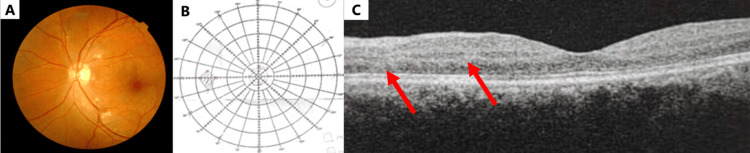
Left eye on day 7 of treatment. (A) Fundus photograph. (B) Bjerrum visual field examination. (C) Optical coherence tomography (OCT) of the macula (red arrows).

By day 7 of treatment, further improvement in the left eye was observed after a three-day course of intravenous methylprednisolone (1 g once daily), followed by oral prednisolone 60 mg once daily. Fundus photography revealed minimal CWS in the peripapillary region and along the arcuate, as well as very minimal dot-blot hemorrhages involving the macula (Figure 3A). Bjerrum visual field test showed a smaller central scotoma (Figure 3B). Subtle PAMM and retinal thickening seen on OCT macula (red arrows) (Figure 3C).

**Figure 4:**
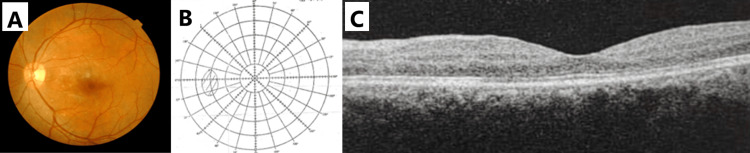
Left eye on day 14 of treatment. (A) Fundus photograph. (B) Bjerrum visual field examination. (C) Optical coherence tomography (OCT) of the macula.

The central scotoma resolved within two weeks; however, fundus abnormalities such as a minute CWS at the peripapillary and inferior arcuate were still seen (Figure 4A). Bjerrum visual field test and OCT macula were normal (Figures 4B-4C). Left visual acuity at day 14 of treatment was 6/6.

**Figure 5:**
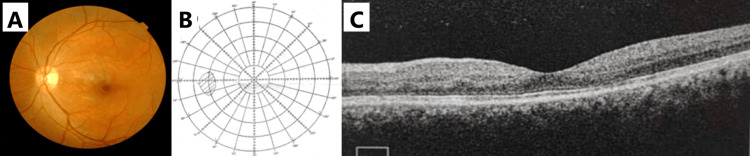
Left eye, four weeks post-trauma. (A) Fundus photograph. (B) Bjerrum visual field examination. (C) Optical coherence tomography (OCT) of the macula.

At four weeks post-trauma, visual acuity improved to 6/5, with normal fundus seen over the left eye (Figure 5A). No central scotoma documented on Bjerrum visual field test (Figure 5B), and there was complete resolution of PAMM and retinal thickening on OCT macula (Figure 5C).

## Discussion

The diagnosis of Purtscher’s and Purtscher-like retinopathies is clinical, with a presentation that usually includes sudden vision loss of variable severity, hours to days after the causal pathology. Fundoscopic signs include CWS and intraretinal hemorrhages, described in 83-92% of a series of cases. Purtscher flecken are pathognomonic but occur in only 50% of cases [6].

Unilateral or asymmetric bilateral posterior TON is a clinical diagnosis that should be considered when visual loss following trauma cannot be explained by other ocular injuries, and there is an RAPD even though the fundus appears normal. Bilateral injury can result in the absence of an RAPD, suggesting that the nerves are comparably damaged [3].

Both pathologies can give poor vision, but the onset can be varied. TON can have immediate visual loss; however, delayed visual loss is documented in 10% of cases [7]. While in Purtscher’s retinopathy, presentation includes sudden vision loss of variable severity, hours to days after the causal pathology, with bilaterality present in up to 60% of cases [2].

In 2013, Miguel et al. reported that 93% of Purtscher’s retinopathy cases have central scotoma [8], which may be caused by a vaso-occlusive process in the choroid and optic nerve and ischemia of the optic nerve [9]. In TON, the visual field is severely compromised at baseline when it is compared with that of normal subjects, but a comparison of baseline visual field and long-term follow-up visit shows a significant improvement in visual field extension [7]. There is no specific visual field defect in patients with TON that has been reported; however, arcuate, central, and hemianopic field defects may be seen [7].

OCT in the acute phase of Purtscher’s retinopathy reveals inner retinal hyperreflectivity at the Purtscher flecken and CWS. Intraretinal and subretinal fluid and retinal thickening may be associated. PAMM, characterized by hyperreflectivity of the inner nuclear layer due to ischemia of the intermediate and deep retinal capillary plexus, might be a feature in the acute phase. Features in the late phase include retinal thinning and photoreceptor loss [4]. TON causes acute axonal loss with severe vision loss [10], with a reduction in circumpapillary retinal nerve fibre layer and ganglion complex cell thickness starting at two weeks after trauma and plateaued at 20 weeks in all cases [11].

In Purtscher’s retinopathy, without treatment, spontaneous visual recovery is seen in 50% of the eyes with improvement of at least two Snellen lines within six months [6], and in another study, late visual recovery was described in a patient who received megadose steroid treatment three weeks after the trauma [12]. It has been proposed that the use of megadose steroids may stabilize the damaged cellular membrane in neuronal tissue, and thereby enable a degree of recovery from tissue insult [13]. It can also block the formation of complement-activated leukocyte aggregation and inhibit the production of oxygen-free radicals [14]. Steroids are thought to exert a neuroprotective effect following trauma, because of antioxidant properties and inhibition of free radical‐induced lipid peroxidation [15].

Visual outcome in Purtscher’s retinopathy is variable. Normal vision may not be recovered in patients with severe initial injury, in whom optic atrophy and macular infarction may persist in a late phase [16]. In the International Optic Nerve Trauma Study (IONTS), there is a relatively high rate of spontaneous visual recovery in TON, and there is no convincing evidence that steroids or surgical optic nerve decompression provides any additional benefit over conservative management alone [15].

In this case, the patient responded very well to high-dose intravenous and oral steroids with a favorable outcome. His visual acuity, visual field, and retinal layers have significantly improved with the treatment.

## Conclusions

Clinical evaluation is crucial for diagnosing indirect trauma cases, as similar presentations can lead to confusion. However, advanced technology like OCT can aid in providing precise diagnoses. OCT imaging is very helpful in diagnosing Purtscher's retinopathy, which may overlap with indirect TON. We recommend using OCT to analyze trauma cases for a better understanding of the ocular pathologies in these patients.
